# Technical note: SpekPy Web—online x‐ray spectrum calculations using an interface to the SpekPy toolkit

**DOI:** 10.1002/acm2.14301

**Published:** 2024-02-16

**Authors:** Robert Vorbau, Gavin Poludniowski

**Affiliations:** ^1^ Medical Radiation Physics and Nuclear Medicine Karolinska University Hospital Stockholm Sweden; ^2^ Department of Clinical Science Intervention and Technology Karolinska Institutet Huddinge Sweden

**Keywords:** half‐value‐laye, software, x‐ray spectrum

## Abstract

Knowledge of the photon spectrum emitted from an x‐ray tube is frequently needed in imaging and dosimetry contexts. As the spectrum characteristics are influenced by several parameters and routine measurement of a spectrum is often impractical, a variety of software programs have been developed over the decades for convenient calculations. SpekPy is a state‐of‐the‐art software package containing several spectrum models, and was created to estimate photon spectra originating from x‐ray tubes using a small set of input parameters (e.g., anode material, anode angle, tube potential, filtration, etc.). SpekPy is distributed as a Python toolkit and is available free of charge. The toolkit does, however, lack a graphical user interface and a user is required to write a Python script to make use of it. In this work this limitation is addressed by introducing a web application called SpekPy Web: a graphical user interface together with an application programmable interface (API). These developments both make the SpekPy spectrum models accessible to a broader set of users and increases the ease of use for existing users. SpekPy Web is hosted at: https://spekpy.smile.ki.se. The functionality of the software is demonstrated, using its API, by estimating first half‐value layers (HVLs) for 15 standard beam qualities from the International Bureau of Weights and Measures (BIPM). The estimated HVLs were found to all be within 3.5% agreement when compared to experimental values, with an average calculation time of 2.5 s per spectrum.

half‐value‐layer, software, x‐ray spectrum

## 1 INTRODUCTION

Estimation of the x‐ray spectra from x‐ray tubes—or quantities derived from such spectra—are pre‐requisites for many calculations in Medical Physics. This is true across many modalities and in both imaging and dosimetry contexts.^1^ Experimental determination of x‐ray spectra is possible,^2^, ^3^ but often impracticable and therefore an array of spectral models and software tools have been developed to fulfil the needs of the scientific community.^4^ Such software is typically distributed as a stand‐alone application,^5^, ^6^ or as a software “toolkit” that requires some experience in a specific programming language.^7^, ^8^ Both approaches have merits, but both require the installation of software. A third, but more rarely adopted option, is to provide a web service in the form of an application that can be accessed via an internet connection, on a desktop or mobile device. This provides the possibility of a graphical user interface (GUI) without a local installation of modelling software.

SpekPy is an open‐source software toolkit developed in the Python programming language.^9^, ^10^ The package allows the user to estimate x‐ray beam spectra and related quantities using some of the most advanced models available. It is worth noting that it is limited to modeling the primary beam and does not simulate secondary processes outside of the target anode, for example, scatter and atomic relaxations from filter materials. SpekPy has become widely used since its release (see standard bibliometric databases). Since SpekPy is a toolkit, however, the user is required to write a short Python program. For users familiar with Python, this is not an issue and even preferable, but for users inexperienced with Python or programming in general, it can be an obstacle. To make it easier for users without programming experience, or users that prefer to work with a GUI or another programming language, we have created SpekPy Web. Building on the SpekPy toolkit, this is hosted as a web service (https://spekpy.smile.ki.se). Any user with an internet connection and a web browser can freely use the web application. No additional software besides a web browser is required.

A further use case for SpekPy Web is for experienced programmers who do not wish to perform a local installation of a toolkit when integrating spectrum calculations in their own software. The web application fulfils this need with the provision of an application programming interface (API), allowing a user to write code to send a request to and receive data from the web service programmatically.

This technical note introduces SpekPy Web and demonstrates its use and capabilities via web browser and API. In addition to the web service, the source code to the application is available free‐of‐charge from the authors, upon request, under the GNU GPLv3 open‐source license.

## 2 METHODS

### 2.1 Graphical user interface and options

**FIGURE 1:**
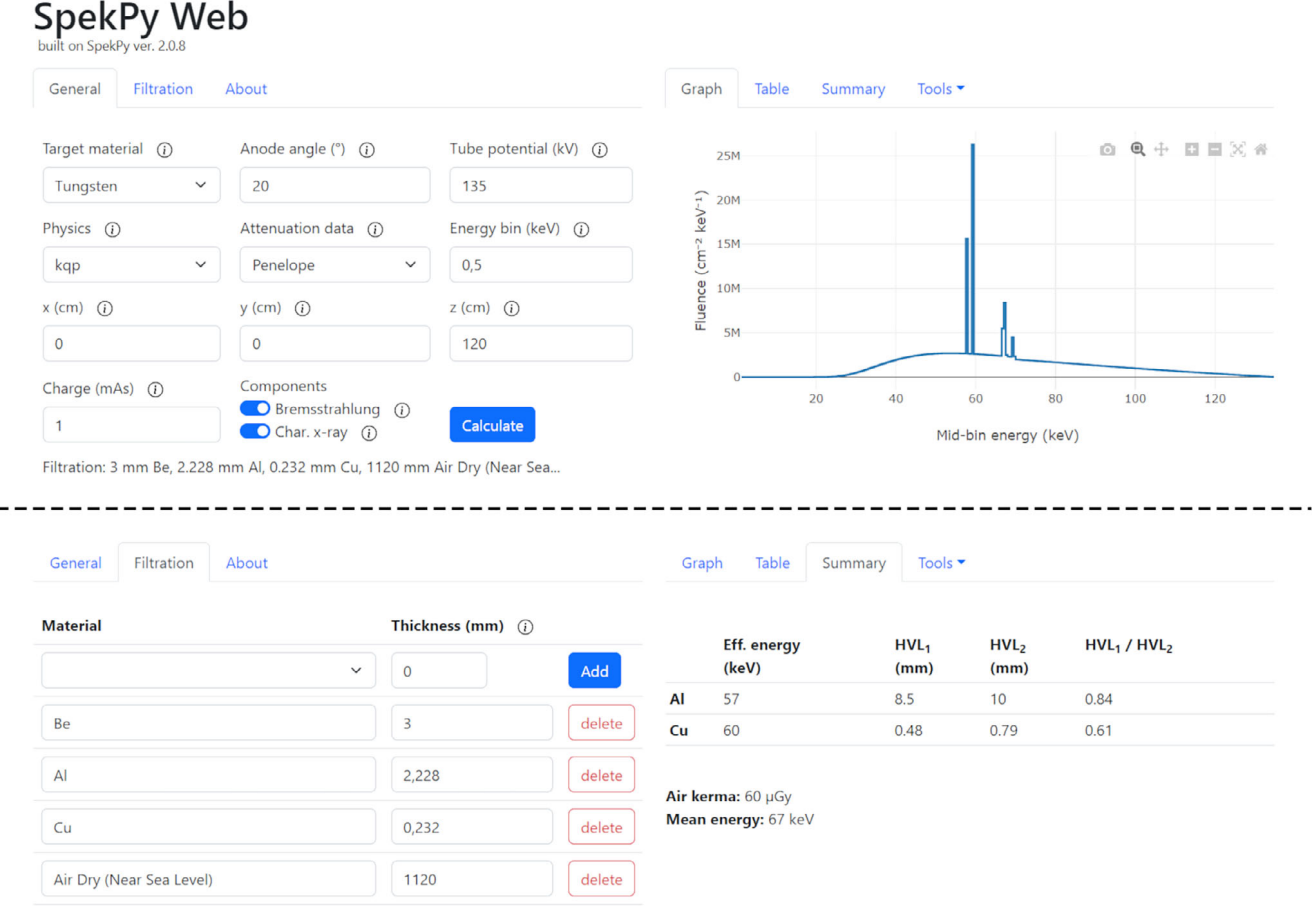
Screenshot of SpekPy Web. The input parameters correspond to calculation of the BIPM‐135 spectrum. The upper panel of the figure illustrates the “General” tab of the user interface; the lower panel illustrates the “Filtration” tab.

SpekPy Web is accessed as a regular web page and runs in a web browser. The layout of the SpekPy Web GUI is divided in two sections, with one section containing the input parameters such as (but not limited to) target material, anode angle and tube potential. Among the input parameters, the user can also specify the desired filtration. The filtration can consist of multiple materials, each with a desired thickness. The material database consists of 445 materials, including all elements from hydrogen (Z = 1) up to uranium (Z = 92), but also materials such as water, common phantom materials, and tissues. An example of how the input parameters are entered in the GUI is shown in Figure 1. In this example the application is set up for calculation of the BIPM‐135 spectrum^11^ using the “kqp” physics model.^12^ The other section of the GUI contains the calculation results, i.e., the resulting spectrum, presented both as a graphical representation and in tabular form. The spectrum table can be extracted as plain text (with tab separated values). Characteristics of the resulting spectrum such as half‐value layers (HVL), and mean and effective energies are also presented in this section of the GUI. Once a spectrum calculation has been made, the web application allows for further analysis. This analysis includes determination of the amount of material needed to reach a desired HVL or the amount of material needed to reduce the air kerma to a desired amount. Any of the filtration materials can be selected in these computations.

### 2.2 Web application architecture

**FIGURE 2:**
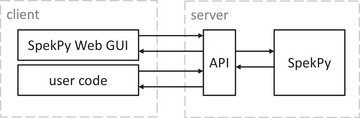
Diagram describing the architecture of SpekPy Web. The SpekPy Web GUI and the user code is running on the client side, while the SpekPy Web API and SpekPy is running on the server side. HTTP messages encoded in JSON format are exchanged between the client and server side through the API. The calculation results are generated by SpekPy. The arrows pointing to right indicate requests and arrows pointing to the left indicate responses.

The SpekPy Web GUI runs in a web browser on the client side. The GUI is an HTML/JavaScript application so all modern web browsers can run it without any additional software installed. When the user presses the “Calculate” button after inserting the desired input parameters, a calculation request is generated by the client and sent to the server using an API that has been developed. The server side handles the request and calculates the spectrum using the SpekPy toolkit. When the computation is finished, the result is sent back to the client using the API, and the results are presented to the user in the web browser. Most of the interactivity on the client side, including sending requests and retrieving responses, is done using the *jQuery* JavaScript library (The OpenJS Foundation). The architecture of the web application is illustrated in Figure 2.

The API used by the web application is made public so that calculation requests are not restricted to the calls implemented in the SpekPy Web GUI. User created code can also request calculations directly through the API. The API feature is aimed at more technically oriented users and requires some understanding of http communication. The requests and responses are sent as HTTP messages with the request and response content encoded in JSON format. The exchange of HTTP messages is illustrated in Figure 2. Software libraries for sending/retrieving HTTP messages and reading/writing JSON are available in many programming languages and can be decided by the user depending on the use case or personal preference. Programming languages with such libraries include both Python (Python Software Foundation) and MATLAB (MathWorks Inc.). The entire API was built using the Python package, *Flask* (The Pallets Project). A summary of the API functionality is shown in Table 1.

**TABLE 1:** Summary of the API functionality

relative URL	HTTP method	explanation
/api/calculate‐spectrum/	POST	Requests a spectrum calculation. Returns a spectrum together with some spectrum characteristics.
/api/calculate‐material‐thickness‐hvl​/	POST	Requests a calculation for the material thickness needed to reach a desired HVL. Returns the material thickness.
/api/calculate‐material‐thickness‐fraction/	POST	Requests a calculation for the material needed to reduce the air kerma to a desired fraction. Returns the material thickness.
/api/filtration‐materials/	GET	Returns a list of all the available filtration materials.
/api/physics‐models/	GET	Returns a list of all available physics models.
/api/target‐materials/{physics_model}/	GET	Returns a list of all available target materials for the specified physics model.
/api/kv‐range/{physics_model}/{target_material}/	GET	Returns the tube potential range available for calculations for the specified physics model and target material.
/api/docs/	GET	Returns a webpage containing the API documentation.

To get the absolute URL, prepend https://spekpy.smile.ki.se to the relative URL. The curly brackets indicate a placeholder to be replaced with suitable a text string. For details about the API see https://spekpy.smile.ki.se/api/docs.

### 2.3 Software evaluation: test case

The software was evaluated by comparing HVL values calculated using SpekPy Web (SpekPy version 2.0.8) to experimentally determined values from BIPM (International Bureau of Weights and Measures)^11^ for 15 standard beam qualities, all with a tungsten anode. The spectrum parameters used for the HVL calculation are listed in Table 2. The computation times for the HVL calculations were also estimated by running each calculation ten times and recording the mean computation time. All computations were performed programmatically using the API. The Python script created for this serves as an illustration of how the API can be used and is therefore provided as Supplemental material. There exist many useful packages for Python that can be used together with the API, but to keep the provided script simple, effort has been made minimize the use of external packages.

**TABLE 2:** Input parameters used in the HVL calculations

		filtration			
name	tube voltage (kV)	Al (mm)	Cu (mm)	Mo (mm)	air (mm)
30	30			0.06	500
25	25	0.372			500
50b	50			0.06	500
50a	50			0.06	500
23 M	23	0.208			500
25 M	25			0.06	500
28 M	28			0.06	500
30 M	30			0.06	500
35 M	35			0.06	500
40 M	40	3.989			500
50 M	50	1.008			500
100	100	3.431			1200
135	135	2.228	0.232		1200
180	180	2.228	0.485		1200
250	250	2.228	1.57		1200

For the x‐ray tube modeled in these calculations (Comet MXR 320 26), the anode is made of tungsten with 20‐degree anode angle; the exit window is made of 3.0 mm beryllium.

## 3 RESULTS

Table 3 presents the results of the test case evaluation. The differences between the estimated HVL values and the reference values ranged from 1.1% to 3.5% (average 1.6%), with all estimated values being lower than the reference values and only one case where the difference exceeded 2.0%. The agreement between the estimated and the reference values is similar to previously published values.^4^, ^10^ The computation times ranged from 1.4 to 6.5 s (average 2.5 s). The computations involving beam qualities with higher tube voltage tend to result in longer computation times compared to beam qualities with lower tube voltage. This is due to the increasing number of energy bins, as the energy bin width (0.5 keV) was kept constant for all computations.

**TABLE 3:** Calculated HVL values using the Spekpy Web API

	HVL (mm Al)		HVL (mm Cu)			
name	reference	estimated	reference	estimated	difference (%)	time (s)
30	0.169	0.166			−1.8	1.7
25	0.242	0.238			−1.8	1.5
50b	1.017	1.005			−1.2	2.1
50a	2.262	2.225			−1.6	2.0
23 M	0.332	0.326			−1.7	1.4
25 M	0.342	0.338			−1.3	1.5
28 M	0.355	0.350			−1.3	1.6
30 M	0.364	0.359			−1.4	1.6
35 M	0.388	0.383			−1.3	1.7
40 M	0.417	0.412			−1.3	1.8
50 M	0.489	0.484			−1.1	2.1
100	4.030	3.949	0.149	0.147	−2.0/−1.1^a^	3.2
135			0.489	0.481	−1.7	3.9
180			0.977	0.960	−1.8	5.0
250			2.484	2.397	−3.5	6.5

Measured HVL values from BIPM^11^ are used as reference. Differences between the estimated values and the reference values are presented together with the computation times recorded when using the API.

^a^ The left value corresponds to difference in HVL expressed in terms of aluminum thickness and the right value corresponds to copper thickness.

## 4 CONCLUSION

A web application called SpekPy Web has been created to make use of the SpekPy toolkit more widely accessible for x‐ray spectrum calculations. The GUI and API were demonstrated in a realistic use case by estimating the first half‐value layers for a set of standard beam qualities produced from x‐ray tubes with a tungsten anode. The agreement between the estimated and the reference values was found to be within 3.5%. Any user with a desktop computer, a laptop or mobile device with an internet connection can use the web application without registration or charge. No additional software besides a web browser is required.

## AUTHOR CONTRIBUTIONS

Gavin Poludniowski was responsible for the conception of the work. Both Robert Vorbau and Gavin Poludniowski designed the user interface to the application. Robert Vorbau solely developed and implemented the software and both authors tested it. Both Robert Vorbau and Gavin Poludniowski co‐authored the manuscript, with Robert Vorbau leading on the authorship.

## CONFLICT OF INTEREST STATEMENT

The authors declare no conflicts of interest.

## Supporting information

Supporting Information
